# Contribution of CTCF binding to transcriptional activity at the *HOXA* locus in *NPM1*-mutant AML cells

**DOI:** 10.1038/s41375-020-0856-3

**Published:** 2020-05-12

**Authors:** Reza Ghasemi, Heidi Struthers, Elisabeth R. Wilson, David H. Spencer

**Affiliations:** 1 Division of Oncology, Department of Medicine, Section of Stem Cell Biology, Washington University School of Medicine, St. Louis, MO USA; 2 McDonnell Genome Institute, Washington University, St. Louis, MO USA

**Keywords:** Cancer genomics, Acute myeloid leukaemia, Cancer epigenetics

## Abstract

Transcriptional regulation of the *HOXA* genes is thought to involve CTCF-mediated chromatin loops and the opposing actions of the COMPASS and Polycomb epigenetic complexes. We investigated the role of these mechanisms at the *HOXA* cluster in AML cells with the common NPM1c mutation, which express both *HOXA* and *HOXB* genes. CTCF binding at the *HOXA* locus is conserved across primary AML samples, regardless of *HOXA* gene expression, and defines a continuous chromatin domain marked by COMPASS-associated histone H3 trimethylation in *NPM1*-mutant primary AML samples. Profiling of the three-dimensional chromatin architecture in primary AML samples with the NPM1c mutation identified chromatin loops between the *HOXA* cluster and loci in the *SNX10* and *SKAP2* genes, and an intergenic region located 1.4 Mbp upstream of the *HOXA* locus. Deletion of CTCF binding sites in the *NPM1*-mutant OCI-AML3 AML cell line reduced multiple long-range interactions, but resulted in CTCF-independent loops with sequences in *SKAP2* that were marked by enhancer-associated histone modifications in primary AML samples. *HOXA* gene expression was maintained in CTCF binding site mutants, indicating that transcriptional activity at the *HOXA* locus in *NPM1*-mutant AML cells may be sustained through persistent interactions with *SKAP2* enhancers, or by intrinsic factors within the *HOXA* gene cluster.

## Introduction

The *HOX* genes encode developmentally regulated transcription factors that are highly expressed in acute myeloid leukemia (AML) and are important drivers of malignant self-renewal in this disease. Previous studies have shown that expression of HOX family members in AML is nearly always restricted to specific genes in the *HOXA* and/or *HOXB* clusters (*HOXC* and *HOXD* genes are rarely expressed), and that expression patterns correlate with recurrent AML mutations [1]. *HOX* expression is most closely associated with AMLs with *MLL* rearrangements, which exclusively express *HOXA* genes, and AMLs with the recurrent NPM1c mutation, which nearly always express both *HOXA* and *HOXB* genes [1]. The high prevalence of *NPM1* mutations make the combined *HOXA/HOXB* expression pattern the most common *HOX* phenotype in AML patients. However, the regulatory mechanisms that drive this expression pattern are poorly understood.

Studies of *HOX* gene regulation in model organisms have established that colinear expression of each *HOX* cluster is mediated by COMPASS/*Trithorax* and Polycomb group proteins, which promote gene activation and repression and perform methylation of histone H3 at lysine 4 (H3K4me3) and 27 (H3K27me3), respectively [2, 3]. These regulatory pathways are also involved in *HOX* gene regulation in AML cells, and are best understood for the *HOXA* cluster in AMLs with *MLL* rearrangements. MLL1 (KMT2A) is a component of the COMPASS complex, and MLL fusion proteins bind to the *HOXA* locus in AML cells and recruit the non-COMPASS histone H3 methyltransferase DOT1L, which is required for *HOXA* activation and AML development in *MLL*-rearranged leukemia models [4–7]. Regulatory DNA elements that control three-dimensional chromatin architecture also play a role in *HOXA* gene regulation in AML cells. Specifically, the *HOXA* and *HOXB* clusters contain multiple binding sites for the chromatin organizing factor CTCF, and chromatin conformation experiments suggest these events mediate local chromatin loops in AML cells with *MLL* rearrangements [8]. In addition, heterozygous deletion of a single CTCF binding site in the *HOXA* cluster in *MLL*-rearranged AML cells resulted in altered chromatin structure and reduced *HOXA* gene expression [9]. These studies suggest that MLL fusion proteins directly activate the *HOXA* locus in ways that require specific CTCF binding events or their associated chromatin structures.

While these mechanistic insights have provided valuable information about *HOXA* regulation in *MLL*-rearranged AML, this molecular subtype accounts for <5% of all AML patients and only 25% of AMLs that express *HOXA* genes [1]. Although AMLs with the *NPM1* mutations nearly always express *HOXA* genes, it is unclear whether *HOXA* expression in these cells shares similar regulatory factors and chromatin structures that appear to be critical for *HOXA* expression in *MLL*-rearranged AML cells. In this study, we investigated histone modifications and chromatin interactions at the *HOXA* locus in *NPM1*-mutated AML samples vs. other AML subtypes, and used a *NPM1*-mutant AML cell line model to determine whether CTCF at the *HOXA* locus is required to maintain *HOXA* expression and chromatin structure.

## Materials and methods

### Primary samples and cell lines

Primary AML samples and normal hematopoietic cells were obtained from diagnostic AML and normal bone marrow aspirates, respectively, following informed consent using protocol (201011766) approved by the Human Research Protection Office at Washington University as described previously [10, 11] (Table S1). All experiments with primary AML samples used bulk cells after estimating the leukemic purity [12]. OCI-AML3 cells obtained from the DSMZ cell repository were cultured at 0.5–1 × 10^6^ cell/mL in MEM alpha with 20% FBS and 1% penicillin-streptomycin. NPM1c was verified in the OCI-AML3 line by targeted sequencing and in RNA-seq data from wild type and mutant clones. Kasumi-1 (received as a gift from T. Ley), IMS-M2 (received as a gift from L. Brunetti), and MOLM13 (received as a gift from J. Dipersio) cell lines were cultured in RPMI-1640 with 1% penicillin-streptomycin and FBS (20% for Kasumi-1 and MOLM13, 10% for IMS-M2).

### Statistical analysis

Hypothesis testing was performed using the indicated parametric statistics after verification of normality. Sample sizes for genomic studies using read count data were selected to provide >80% power to detect a fold-change of at least 2.

### ChIP-seq

ChIP-seq was performed using ChIPmentation [13] with the following antibodies: CTCF (2899S), H3K27me3 (9733S), and H3K27ac (8173S) from Cell Signaling Technology and H3K4me3 (ab1012) from Abcam. Sequencing was performed on a NovaSeq 6000 (Illumina, San Diego, CA) to obtain ~50 million 150 bp paired-end reads. Data were analyzed via adapter trimming with trimgalore and alignment to GRCh38 using bwa mem [14]. Normalized coverage for visualization and analysis used the deeptools “bamCoverage” tool [15], and peaks were called with macs2 [16] for CTCF and epic2 [17] for histone marks. Statistical comparisons with DESeq2 [18] used raw fragment counts at peak summits, and visualizations were prepared with Gviz [19].

### Targeted deletion of CTCF binding sites

Deletions were generated using CRISPR/Cas9 with guide RNAs from the UCSC genome browser [20, 21] that overlapped CTCF ChIP-seq peaks (Table S2). Mutagenesis used either inducible Cas9-expressing OCI-AML3 cells (Lenti-iCas9-neo vector; Addgene 85400) with lentiviral sgRNA expression (Addgene 70683), or transfection with Cas9 protein and tracrRNA/crRNA hybrids (Alt-R system, IDT, Coralville, IA). For the latter, RNAs were complexed with Cas9 protein using the manufacturer’s protocol with 1 million cells and 28 μM of Cas9/RNA for either transfection (CRISPRMAX; Thermofisher Scientific, Waltham, MA) or nucleofection (SG Amaxa Cell Line 4D-Nucleofector Kit, Lonza, Basel, Switzerland). Mutation efficiency was assessed in bulk cultures via DNA extraction, PCR with tailed primers (Table S2), and sequencing to obtain 2 × 250 bp reads on an Illumina MiSeq instrument and analysis of minimap2-based alignments [22]. Sorting into 96 well plates via FACS was used for expansion of individual clones. Cells from single wells were screened via direct lysis by proteinase K (P8107S; NEB) in 20 μl of single-cell lysis buffer (10 mM Tris-HCl pH 7.6, 50 mM NaCl, 6.25 mM MgCl_2_, 0.045% NP40, 0.45% Tween-20), PCR, and gel electrophoresis; clones with evidence for deletions were sequenced, and clones with deletions were expanded for analysis.

### RNA analysis

RNA extractions were performed on ~1 million cells with the Quick-RNA MicroPrep Kit (Zymo Research, Irvine, CA). One hundred nanograms of RNA was used for cDNA synthesis (Applied Biosystems, Foster City, CA), and qPCR was done in duplicate in a StepOnePlus PCR System (Applied Biosystems) for *HOXA9* exons 1-2 with a *GUSB* control (IDT). RNA-seq libraries were generated using 300 ng of RNA with the KapaHyper stranded RNA library kit for Illumina (Roche) following the manufacturer’s instructions and then sequenced on a NovaSeq 6000 to obtain ~50 million 2 × 150 bp reads. Reads were trimmed with trimgalore, aligned with STAR [23], and transcript-level expression values in TPM were obtained with stringtie [24].

### In situ Hi-C

Hi-C libraries were prepared as described [25] using 4–5 million cells. Libraries were first assessed by sequencing ~1 million reads on a MiSeq instrument (using metrics recommended by Rao et al. [25]); passing libraries were sequenced to obtain 2 × 150 bp reads on a NovaSeq 6000. Hi-C data were analyzed on GRCh38 with juicer [26]. All analyses used contact matrices (mapping quality >30), and chromatin loops and contact domains identified using HICCUPS and arrowhead, respectively [26]. Loops from wild-type and mutant OCI-AML3 cells were merged using bedtools “pairToPair” function [27] with 5000 bp overlap. Pairwise and joint comparisons of chromatin loops were performed with hicCompare [28] and multiHicCompare [29], respectively, using raw contact counts from chromosome 7 obtained from the juicer hic file at 25 kbp resolution. Statistical comparisons used chromosome-wide FDR corrections for multiple hypothesis testing. Visualizations used the HiTC [30], GenomicInteractions, and Gviz R packages.

## Results

### CTCF defines dynamic chromatin domains at the *HOXA* locus in primary AML cells

**Fig. 1:**
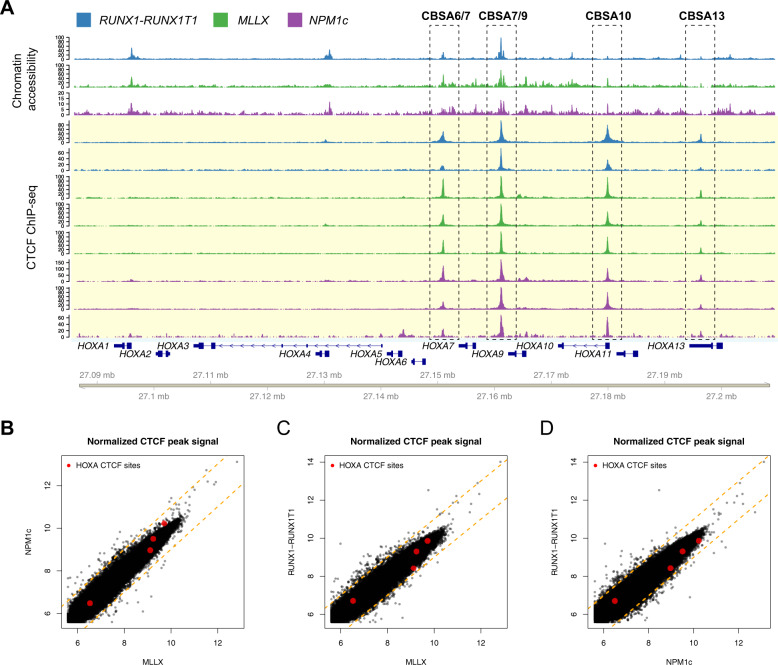
CTCF is bound to chromatin-accessible sites at the *HOXA* locus in primary AML samples. **a** Chromatin accessibility by ATAC (top tracks highlighted in white; *N* = 1 from each mutation category, from ref. [1]) and ChIP-seq for CTCF (bottom tracks highlighted in yellow) from primary AML samples with either t(8;21) creating the *RUNX1-RUNX1T1* gene fusion (blue; *N* = 2), *MLL* rearrangements (green; *N* = 3), or a normal karyotype and *NPM1* mutation (purple; *N* = 3). CTCF sites CBSA6/7, CBSA7/9, CBSA10, and CBSA13 are indicated by the dashed boxes. **b**–**d** Scatter plots comparing the CTCF peak summit counts between each AML type in log2 normalized read counts (mean of three samples for NPM1c and *MLL*-rearranged samples, mean of two for *RUNX1-RUNX1T1*). Red points indicate ChIP-seq signal for the four CTCF binding sites highlighted in **a**, which are similar across AML samples. Dashed orange lines indicate a twofold change between the samples.

We previously showed that specific regions in the *HOXA* cluster have accessible chromatin in primary AML samples that coincide with CTCF binding sites in other human cell types [1]. To confirm the presence of CTCF at these loci in primary AML cells, we performed CTCF ChIP-seq on primary AML patients with the NPM1c insertion mutation in *NPM1*, t(9;11) and t(11;19) *MLL* rearrangements, and t(8;21) creating the *RUNX1-RUNX1T1* gene fusion, which displayed the expected *HOXA* and *HOXB*, *HOXA* only, and no *HOX* expression patterns, respectively (Table S1 and Fig. S1A). This confirmed CTCF binding in chromatin-accessible regions between *HOXA6* and *HOXA7* (“CTCF binding site CBSA6/7”), and *HOXA7* and *HOXA9* (“CBSA7/9”), near the transcriptional start site of *HOXA10* (“CBSA10”), and in the 5′ UTR of *HOXA13* (“CBSA13”) (Fig. 1a). All four CTCF peaks were present in all AML samples regardless of *HOXA* expression status, and quantification of the CTCF signal demonstrated similar occupancy across the AML types (Fig. 1b–d).

**Fig. 2:**
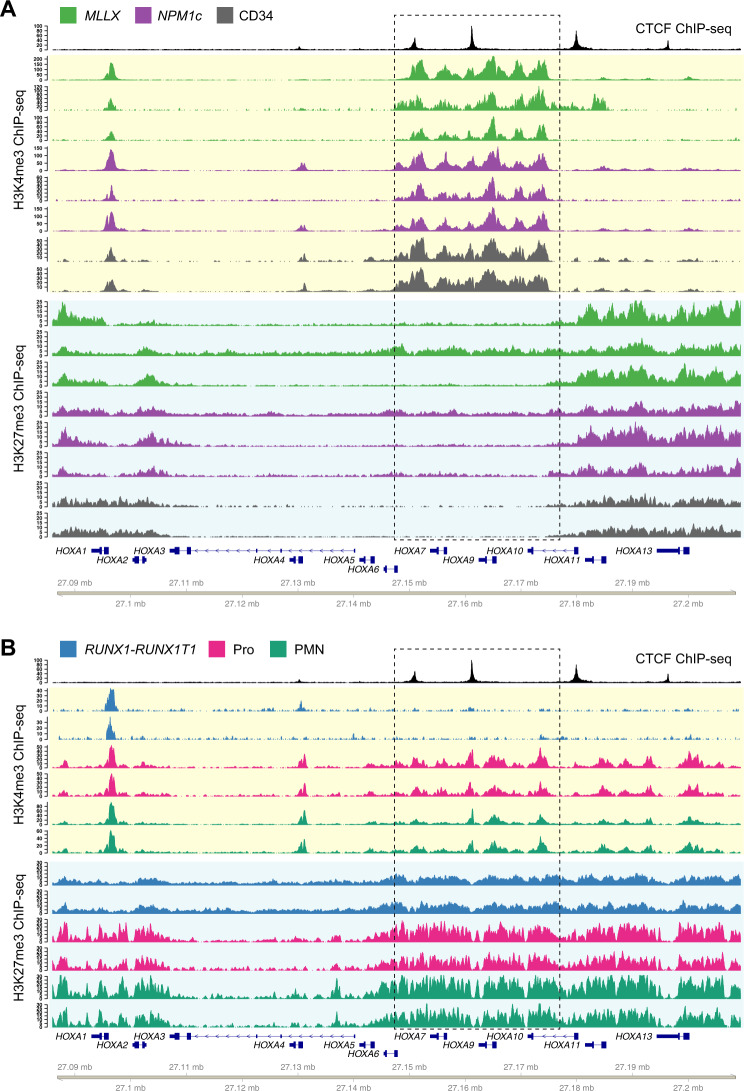
CTCF defines chromatin boundaries in AML samples and normal CD34^+^ cells with *HOXA* gene expression. **a** Top track shows CTCF ChIP-seq from a *NPM1*-mutant primary AML sample. Tracks highlighted in yellow show ChIP-seq for H3K4me3 from primary samples with high *HOXA* expression, including AML samples with *MLL* rearrangements (green; *N* = 3) and *NPM1* mutations (purple; *N* = 3), and primary CD34^+^ hematopoietic stem/progenitor cells (HSPCs) purified from normal donor bone marrow samples (gray; *N* = 2, from GSE104579). Tracks highlighted in blue show H3K27me3 ChIP-seq from the same set of samples. **b** H3K4me3 and H3K27me3 ChIP-seq from primary samples with no *HOXA* expression, including AML samples with t(8;21) creating the *RUNX1-RUNX1T1* fusion (blue; *N* = 2), and normal promyelocytes (CD14−, CD15+, CD16 low; magenta, *N* = 2) and neutrophils (CD14−, CD15+, CD16 high; cyan, *N* = 2) from healthy donor individuals. Dashed box indicates the region of dynamic chromatin that correlates with *HOXA* gene cluster expression.

We next determined whether these CTCF binding events defined chromatin domains in primary AML samples by performing ChIP-seq for H3K4me3 and H3K27me3 to measure active and repressed chromatin, respectively. This identified a region of active chromatin in the center of the *HOXA* cluster that overlapped CBSA6/7 and CBSA7/9, and was conserved in the *MLL*-rearranged and *NPM1*-mutant AML samples, and normal CD34^+^ cells (which also express *HOXA* and *HOXB* genes) (Figs. 2a and S2A). The H3K4me3 signal was continuous across this interval, including non-promoter sequences, and was also marked with H3K27ac in primary AML samples (Fig. S2B). Adjacent regions were marked with repressive H3K27me3 in all AML samples and in CD34^+^ cells, which correlated with the expression levels of the overlapping genes (Figs. S1A, S2A). ChIP-seq using AML samples with the *RUNX1-RUNX1T1* gene fusion and low *HOXA* expression showed that the active chromatin domain is dynamic, with little H3K4me3 signal and increased H3K27me3 in this AML type (Fig. 2b). Similar repressive histone methylation patterns were observed in ChIP-seq data from FACS-purified normal promyelocytes and mature neutrophils [10] that also do not express *HOXA* genes (Figs. 2b and S2A).

### Targeted deletions at the *HOXA* locus eliminate CTCF binding but do not affect viability in *NPM1*-mutant OCI-AML3 cells

**Fig. 3:**
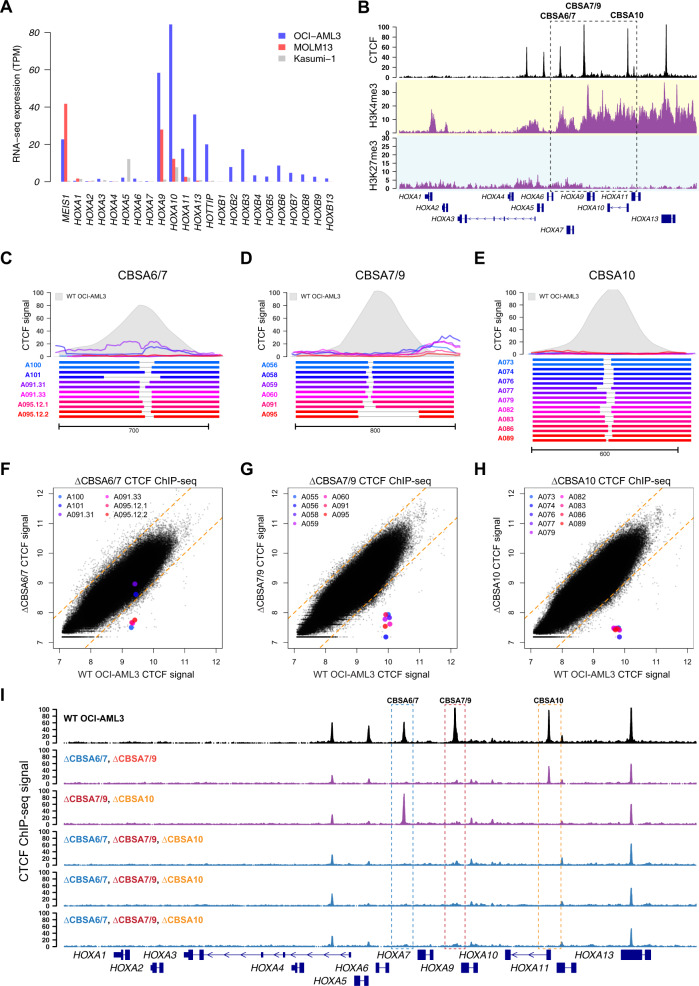
Targeted deletions eliminate CTCF binding in the *NPM1*-mutant OCI-AML3 cell line. **a** RNA-seq expression of *HOXA* and *HOXB* genes in OCI-AML3 cells, which display the canonical mutant *NPM1*-associated *HOXA/HOXB* expression phenotype. Also shown are the *MLL*-rearranged MOLM13 cell line that expresses only *HOXA* genes, and the *RUNX1-RUNX1T1*-containing Kasumi-1 cell line that has low *HOXA* and *HOXB* gene expression. **b** ChIP-seq data from OCI-AML3 cells for CTCF (black), H3K4me3 (highlighted in yellow), and H3K27me3 (highlighted in blue), which show conserved CTCF binding sites and distinct regions of active (H3K4me3) and repressed (H3K27me3) chromatin. **c**–**e** Targeted deletions that disrupt CTCF binding in OCI-AML3 cells at sites CBSA6/7 (in **c**), CBSA7/9 (in **d**), and CBSA10 (in **e**). Bottom panels show allele pairs from homozygous or compound heterozygous deletion mutants at each site; top panels show CTCF ChIP-seq signal from these mutant cell lines (multi-colored lines) compared with wild-type OCI-AML3 cells (in gray). **f**–**h** CTCF ChIP-seq signal (log_2_ normalized read counts) for all CTCF peaks from deletion mutants (Y axis) vs. wild-type OCI-AML3 cells, showing dramatically reduced CTCF ChIP-seq signal in deletion mutants at all three sites, with the exception of clones A101 and A091.31, which only partially eliminates CTCF signal at site CBSA6/7. **i** CTCF ChIP-seq tracks from double (in purple) and triple mutants (in blue), generated via sequential-targeted deletion experiments. CTCF ChIP-seq from wild-type OCI-AML3 cells is shown in black at the top for reference.

We sought to determine whether loss of these DNA elements has functional consequences in *NPM1*-mutant AML cells. To this end, we used the OCI-AML3 cell line with a canonical *NPM1* insertion mutation and that expresses *MEIS1* and both *HOXA* and *HOXB* genes [31]. This pattern was not observed in cell lines with other mutation-associated *HOX* expression phenotypes, including *MLL*-rearranged MOLM13 cells that expressed only *HOXA* genes, and the *RUNX1-RUNX1T1-*containing Kasumi-1 cell line, which had low *HOX* expression (Fig. 3a). ChIP-seq for CTCF using OCI-AML3 cells identified the four conserved CTCF sites observed in primary AML samples (Figs. 3b and S3A), as well as two smaller peaks in the anterior *HOXA* cluster. ChIP-seq for H3K4me3 and H3K27me3 demonstrated an active chromatin domain between *HOXA9* and *HOXA13* (Fig. 3b), which was consistent with the patterns of gene expression in this cell line, but different from chromatin domain boundaries in primary AML samples. However, the histone modifications between CBSA7/9 and CBSA10 were shared between OCI-AML3 cells and *NPM1*-mutated primary AML samples, suggesting that some of the regulatory activities in this region are conserved between primary AML samples and OCI-AML3 cells.

We next used CRISPR/Cas9-mediated editing to delete the three conserved CTCF binding sites in OCI-AML3 cells (CBSA6/7, CBSA7/9, and CBSA10). The resulting mutations did not appreciably alter markers of cell maturation in the edited cells (Fig. S3B, C), and the deletion frequency at CBSA7/9 and CBSA10 remained stable after 7 and 14 days; CBSA6/7 deletions were also stable, although the lower deletion efficiency for this site means that subtle fitness defects cannot be excluded (Fig. S3D). Single cells were expanded and screened for deletions, which identified at least five individual clonal lines with homozygous deletions at each site (Table S3), with deletions as small as 9 bp sufficient to eliminate nearly all CTCF ChIP-seq signal (Fig. 3c–h). Additional experiments using single deletion mutants resulted in ten doubly homozygous mutants and seven triple mutants with homozygous deletions at all three sites (Fig. 3i). None of the mutant OCI-AML3 clones showed overt defects in cell growth (Fig. S3E), despite complete loss of CTCF binding in the posterior *HOXA* cluster.

### CTCF binding is not required for maintenance of *HOXA* gene expression or chromatin boundaries in *NPM1*-mutant AML cells

**Fig. 4:**
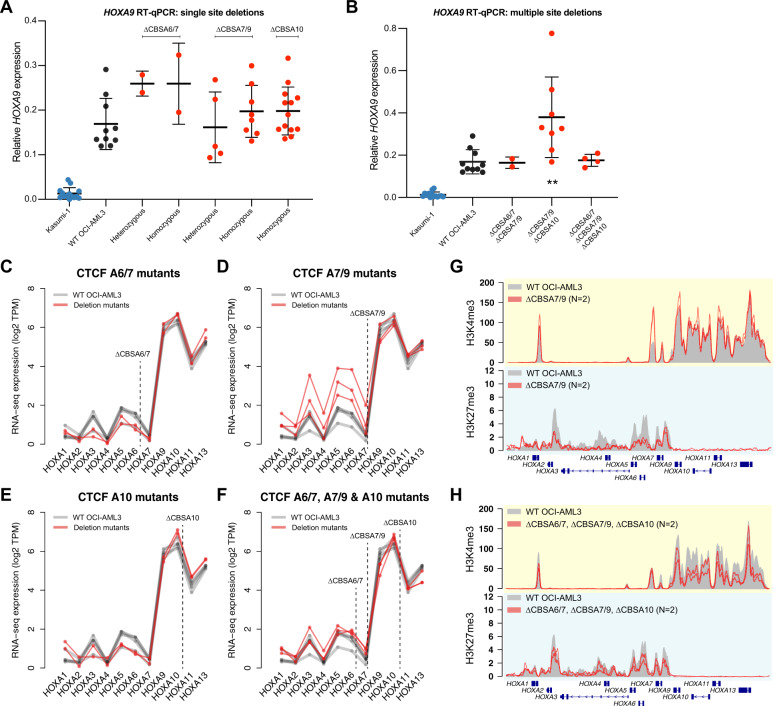
CTCF binding is not required to maintain gene expression or chromatin boundaries in the *HOXA* gene cluster. **a** RT-qPCR for *HOXA9* in single mutants with heterozygous or homozygous deletions at CBSA6/7, CBSA7/9, or CBSA10. *HOXA9* expression from the Kasumi-1 cell line is shown in blue as a no-*HOXA9* expressing control. No statistically significant differences were identified between wild-type OCI-AML3 and homozygous mutants (Bonferroni-corrected *P* > 0.05 using a two-sided unpaired *T*-test for all comparisons). **b** RT-qPCR for *HOXA9* in double and triple mutants at the CTCF binding sites indicated. Kasumi-1 cells are included in blue as in **a**. ** denotes a Bonferroni-corrected *P* < 0.01 between wild-type OCI-AML3 and CBSA7/9-CBSA10 double mutant clones using a two-sided unpaired *T*-test. **c**–**f** RNA-seq expression of all *HOXA* genes in single mutants (**c**–**e**) and triple mutants (**d**). Expression level is shown in log_2_ transcripts per million (TPM), with lines connecting expression values derived from the same mutant clones and/or the same RNA-seq experiment (for wild-type OCI-AML3 cells). **g** ChIP-seq for H3K4me3 and H3K27me3 in deletion mutants lacking CTCF at site CBSA7/9 (in red; *N* = 2). Mean ChIP-seq signal from wild-type OCI-AML3 cells (*N* = 2) is shown in gray. **h** Mean ChIP-seq for H3K4me3 and H3K27me3 in triple mutants (*N* = 2), with ChIP-seq from wild-type cells shown in gray, as in **g**.

We selected 45 OCI-AML3 mutants for *HOXA9* expression analysis via RT-qPCR to assess whether loss of CTCF binding affected *HOXA9* expression. Surprisingly, *HOXA9* expression was essentially unchanged in all deletion lines, with little evidence for consistent reduction in expression in the mutant clones, and no trend toward decreased expression between wild-type, heterozygous, and homozygous single mutants (Fig. 4a) or multiply mutated clones (Fig. 4b); in fact, *HOXA9* expression was increased in some mutants. Analysis via RNA-seq showed modest increases in the expression of the anterior *HOXA* genes *HOXA1*-*HOXA7* in lines with CBSA7/9 deletions, and subtle increases in the posterior *HOXA9-HOXA13* genes when CBSA10 was deleted (Figs. 4d, e and S4A). However, most mutant lines showed few expression changes. Furthermore, a heterozygous single nucleotide polymorphism in the *HOXA9* gene showed balanced expression of both alleles in wild-type OCI-AML3 cells, and in all mutant lines, except for two with large mutations that involved the posterior *HOXA* cluster (Fig. S4B).

ChIP-seq for H3K4me3 and H3K27me3 was also performed on multiple mutants to determine whether loss of CTCF binding altered chromatin boundaries. H3K4me3 signal was reduced specifically at the CBSA7/9 site, but was otherwise intact. H3K27me3 was also still present, but was modestly decreased across the anterior *HOXA* genes in CBSA7/9 mutants; few changes and no statistically different peaks were present in other mutant lines, including triple mutants (Figs. 4g, h and S4C–G). Other histone modifications were also analyzed, including H3K79me2 and H3K27ac, which were intact in the mutant lines compared with wild-type OCI-AML3 cells (Fig. S4H).

### CTCF deletions result in compensatory *HOXA* chromatin loops

**Fig. 5:**
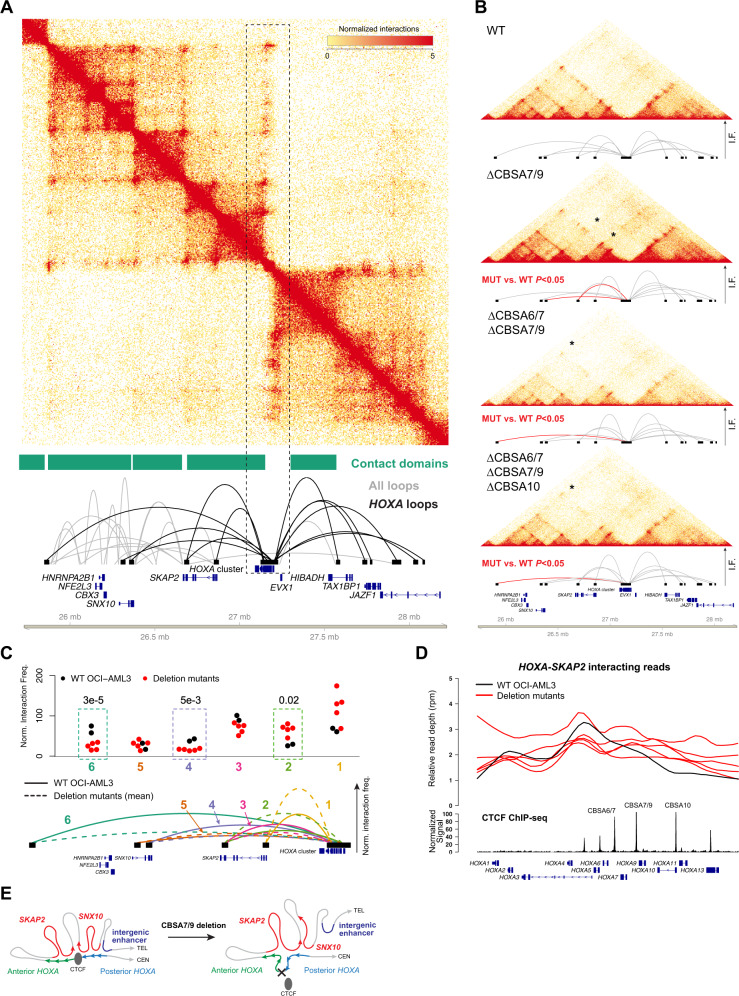
CTCF-mediated chromatin architecture at the *HOXA* locus in *NPM1*-mutant OCI-AML3 cells. **a** Contact matrix, contact domains, and chromatin loops for a 2.5 Mbp region of chromosome 7p that contains the *HOXA* gene cluster from in situ Hi-C using wild-type OCI-AML3 cells. Top panel shows the KR-normalized contact matrix at 5 kb resolution. Tracks below the matrix show the contact domains and statistically supported chromatin loops identified using established methods [26]. Loops shown in black are anchored within the *HOXA* gene cluster. **b** Contacts and *HOXA* region loops from in situ Hi-C from wild-type OCI-AML3 cells and homozygous (biallelic) single mutants at CBSA7/9, double mutants at CBSA6/7 and CBSA7/9, and triple mutants at CBSA6/7, CBSA7/9, and CBSA10. Loops in red indicate statistically significant differences in pairwise comparisons between each mutant and wild-type cells with a chromosome-wide FDR < 0.05. **c** Normalized interaction frequencies for all datasets for six telomeric loops. Top panel shows normalized interaction frequencies for individual Hi-C libraries from wild-type OCI-AML3 cells (*N* = 2), single mutants at CBSA7/9, CBSA6/7-CBSA7/9, and CBSA7/9-CBSA10 double mutants, and two CBSA6/7-CBSA7/9-CBSA10 triple mutant lines (*N* = 5 total mutant lines). Dashed boxes highlight loops that were statistically different in comparisons of all mutant libraries vs. two wild-type OCI-AML3 libraries (chromosome-wide FDR < 0.5). Mean interaction frequencies for chromatin loops from wild-type and mutant lines are shown graphically in the lower panel in solid and dashed lines, respectively, and are numbered so they correspond with the normalized frequencies shown in the top panel. **d** Relative read depth (reads per million) across the *HOXA* locus for reads that interact between *HOXA* and *SKAP2*. Depth for wild-type OCI-AML3 cells is shown in black and deletion mutants are shown in red. CTCF ChIP-seq signal from wild-type OCI-AML3 cells and *HOXA* genes are shown in the bottom tracks. CTCF site deletions result in enhanced interactions between the posterior *HOXA* cluster (genes *HOXA9*-*HOXA13*) with the *SKAP2* gene. **e** Graphical representation of *HOXA* chromatin loops in wild-type OCI-AML3 cells (left) and deletion mutants lacking *HOXA* CTCF binding sites (right).

Given the role of CTCF in regulating chromatin architecture, we used in situ Hi-C [25] to define the chromatin interactions at the *HOXA* locus in wild-type OCI-AML3 cells, CBSA7/9 single mutants, double mutants with deletions of CBSA6/7 and CBSA7/9 or CBSA7/9 and CBSA10, and two triply mutant lines (Table S4). Analysis of the chromatin contacts from these cells identified a mean of 9797 chromatin loops and 5160 contact domains at 10 kbp resolution (ref. [26]; Table S4). The *HOXA* cluster on chromosome 7p was located at a contact domain boundary (Fig. 5a), with centromeric loops connecting *HOXA13* to the promoter of *TAX1BP1* and intronic sequences of *HIBADH* and *JAZF1*. The telomeric loops involved the remaining *HOXA* genes (*HOXA1*-*HOXA11*), which contacted regions in the *SKAP2*, and *SNX10* genes, and an intergenic region 1.4 Mbp upstream with no associated gene annotations. Similar chromatin loops were observed in Hi-C data generated from the *MLL*-rearranged MOLM13 cell line, and in previously reported data from normal human HSPCs [32] (Fig. S5A, B), which both express *HOXA* genes.

We next analyzed Hi-C data from the OCI-AML3 deletion mutants to determine whether loss of CTCF binding altered chromatin architecture at the *HOXA* gene cluster. Although the general contact domain and chromatin loop structure remained intact, loops involving the *HOXA* locus were altered in the deletion mutants (Fig. 5b). Pairwise comparisons of normalized chromosome 7 interaction frequencies between each mutant line vs. wild-type cells identified decreased long-range interactions and increased interactions with the more proximal *SKAP2* gene (Figs. 5b and S5C). These findings were confirmed via statistical analysis of joint normalized contact frequencies from all samples, which demonstrated that mutant cells had significantly reduced interaction frequencies between the *HOXA* cluster and *SNX10* and the intergenic region, while interactions with two *SKAP2* loci that were present in wild-type cells were increased (Fig. 5c and Table S5). To define the specific *HOXA* genes involved in these changes, we mapped the positions of *HOXA-SKAP2* interacting reads within the *HOXA* locus. This showed that *SKAP2*-*HOXA* loops involved the anterior *HOXA* genes and intron 1 of *SKAP2* in wild-type cells (Fig. 5d); however, in mutant cells there were increased interactions between the posterior *HOXA* genes *HOXA9-HOXA13* and intron 11 of *SKAP2* (Fig. 5d). Interacting reads with *SNX10* and the intergenic locus showed the opposite pattern, and were decreased in the posterior *HOXA* cluster (Fig. S5D), indicating that loss of CTCF binding resulted in “spreading” of the proximal *SKAP2* interactions to include the posterior *HOXA* genes (Fig. 5e).

### Posterior *HOXA* chromatin loops correlate with expression and involve potential enhancer loci in the *SKAP2* gene in primary AML samples

**Fig. 6:**
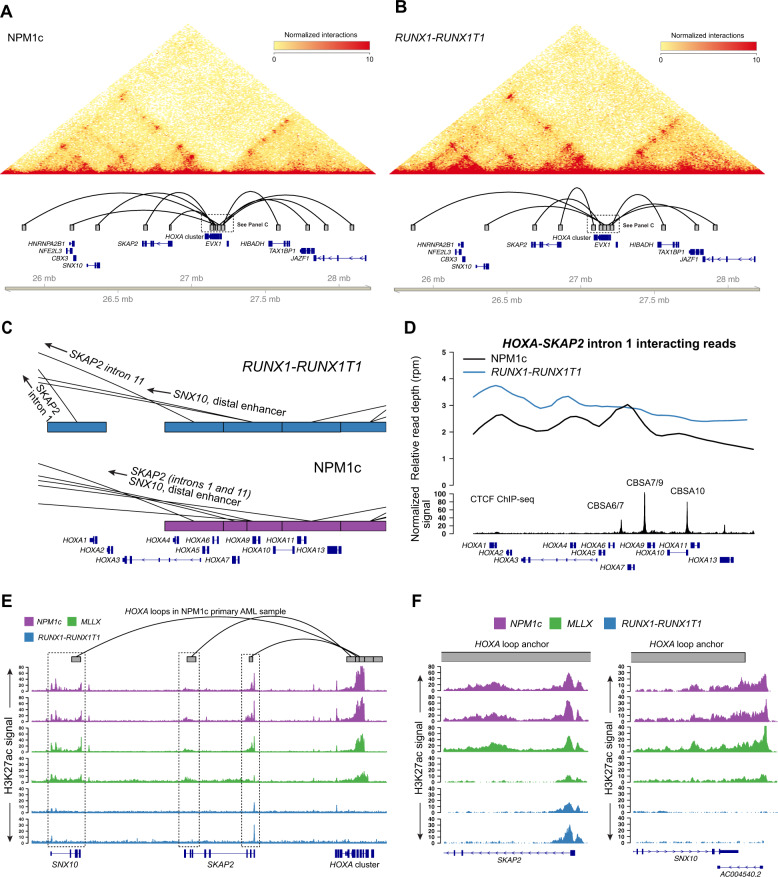
Chromatin interactions with the posterior *HOXA* genes correlate with gene expression in primary AML samples and involve loci with enhancer-associated histone acetylation. **a** Chromatin interactions at chromosome 7p from primary AML sample 507202 with the NPM1c mutation and high *HOXA* gene expression (see Fig. S1A). Heatmap shows the contact matrix for this sample at 10 kbp resolution. Tracks in the lower panel show statistically supported chromatin loops that involve the *HOXA* gene cluster. **b** Chromatin interactions for a primary AML sample with t(8;21)/*RUNX1*-*RUNX1T1* and no *HOXA* gene expression (see Fig. S1A). Panel components are the same as **a**. **c** Focused view of *HOXA* chromatin loops in the *RUNX1*-*RUNX1T1* (top, in blue) and NPM1c (bottom, in purple) primary AML samples, which highlight the differences in chromatin loop structure and the locations of the loop anchors, which were shifted to the posterior *HOXA* cluster in the NPM1c-positive sample. **d** Relative read depth in reads per million of Hi-C reads between the *HOXA* locus and intron 1 of *SKAP2* in the primary samples with NPM1c and *RUNX1-RUNX1T1* black and blue, respectively. Interacting reads in the sample with *RUNX1-RUNX1T1* are localized to *HOXA1* in the anterior *HOXA* cluster, compared with the NPM1c sample where interacting reads where interacting reads map to CBSA7/9 in the posterior *HOXA* locus. **e** Enhancer-associated histone H3 lysine 27 acetylation (H3K27ac) at *HOXA* interacting loci from primary AML samples with and without *HOXA* gene expression (including the samples shown in **a**, **b**). Primary AML samples include patients with NPM1c (purple; *N* = 2 distinct patients; the first track is AML 507202 from **a** and *MLL* rearrangements (t(9;11) and t(11;19)) (green; *N* = 2 distinct patients) with high *HOXA* expression, and samples with t(8;21)/*RUNX1*-*RUNX1T1* (*N* = 2 distinct patients; the first track is AML 275786 from **b**). Regions highlighted in the dashed boxes interact with the *HOXA* cluster in the NPM1c-containing primary sample 507202 (loop track, top), and display H3K27ac signal suggesting they may represent functional genomic elements. **e** High-resolution view of two loop anchor regions in *SKAP2* intron 1 and downstream of the *SNX10* gene, which possess the enhancer-associated H3K27ac mark in *HOXA*-expressing AML samples but not in samples with no *HOXA* expression.

To determine the relationship between transcriptional activity and chromatin loops involving the posterior *HOXA* genes, we performed in situ Hi-C on primary AML samples with the NPM1c mutation and with high *HOXA* expression (AML 507202) and a sample with the *RUNX1-RUNX1T1* gene fusion and low *HOXA* gene expression (AML 275786; Fig. S1A). Hi-C libraries from these samples captured between 284 and 494 million contacts, of which at least 42% were long range (Table S4). These samples displayed similar overall patterns of chromatin interactions in the *HOXA* region, which included loops between the *HOXA* cluster and introns 1 and 11 *SKAP2*, the *SNX10* gene, as well as the distal enhancer locus (Fig. 6a, b). However, there were clear differences in the number of loops, and the specific locations of the loop anchors in the *HOXA* cluster, which correlated with *HOXA* expression. There were more statistically supported chromatin loops in the *NPM1*-mutant AML sample compared with the *RUNX1-RUNX1T1* sample (5 vs. 3), and the region of the *HOXA* cluster that interacted with intron 1 of *SKAP2* involved posterior *HOXA* genes in the NPM1c sample, but was restricted to the anterior *HOXA1* gene in the *RUNX1-RUNX1T1* sample (Fig. 6c). Similar findings were present in Hi-C data from AML cell lines with the same mutations, including OCI-AML3 and IMS-M2 cells with the NPM1c mutation and the Kasumi-1 cell line with the *RUNX1-RUNX1T1* gene fusion, which showed that NPM1c-containing cells had more loops and a shift in the loop anchors toward the posterior *HOXA* cluster (Fig. S6A–C). Further analysis of the interactions between *SKAP2* intron 1 and the *HOXA* cluster in the primary AML samples demonstrated that reads supporting this loop were most abundant near the CTCF binding site CBSA7/9 in the NPM1c sample, compared with the *HOXA1* gene in the sample with *RUNX1-RUNX1T1* (Fig. 6d).

We next analyzed ChIP-seq data for H3K27ac from primary AML samples (including those analyzed with Hi-C above) to determine whether loci that interact with *HOXA* genes may have enhancer properties. Indeed, intron 1 of *SKAP2* (and to some extent intron 11) displayed clear H3K27ac signal in *NPM1*-mutant AML samples, as well as *MLL*-rearranged samples that also express *HOXA* genes (Fig. 6e, f). Loci near the *SNX10* gene also possessed this mark, including a noncoding RNA downstream of the 3′ UTR of *SNX10* (Fig. 6f), but H3K27ac was not present at other regions that formed contacts with the *HOXA* cluster, including the distal intergenic locus (Fig. S6D). Although the enhancer modifications observed at interacting regions had relatively low signal, they were clearly absent in the *RUNX1-RUNX1T1* samples, which lack *HOXA* expression. This provides evidence that these specific regions interact with the *HOXA* cluster in primary AML samples that express *HOXA* genes, and may therefore contribute to *HOXA* gene regulation.

## Discussion

*HOX* transcription factors are drivers of self-renewal in AML cells and are highly expressed in AMLs with *NPM1c* mutations. In this study, we demonstrated that the chromatin organizing factor CTCF is bound equally to specific sites at the *HOXA* locus in *NPM1*-mutant primary AML samples compared with other AML subtypes, and define a dynamic chromatin domain in primary AML samples and normal hematopoietic cells. Targeted deletions in the *NPM1*-mutant OCI-AML3 cell line eliminated CTCF binding, but surprisingly did not disrupt *HOXA* gene expression. This was true when multiple binding sites were deleted individually, or in combination, and was supported by independent mutant clones that showed little evidence for consistent decreases in *HOXA* gene expression, or changes to the histone modifications we measured. However, loss of CTCF binding did result in clear alterations to the chromatin loops involving the posterior *HOXA* genes, including *HOXA9* and *HOXA10*. Specifically, long-range loops were diminished, and were replaced by compensatory interactions with regions of the *SKAP2* gene, which also appear to directly interact with active *HOXA* genes in primary AML samples with the NPM1c mutation. Some of these candidate enhancers have been reported in other studies [33], but have not previously been shown to be active in hematopoietic cells. Further investigation of these sequences, and their associated regulatory proteins, may shed light into factors that promote *HOXA* gene activation in normal and malignant myeloid cells.

The central observation in this study is that CTCF binding at the *HOXA* cluster is not absolutely required for maintenance of *HOXA* expression in *NPM1*-mutated AML cells. Deletion of CTCF site CBSA7/9 was previously shown to affect *HOXA* expression in the *MLL*-rearranged MOLM13 cell line [9]. These discrepant findings may be due to fundamental differences in how *HOX* genes are regulated in *MLL*-rearranged vs. *NPM1*-mutant AML cells. Indeed, the *HOX* expression phenotype of these AML subtypes is strikingly different: *MLL* rearrangements are associated with only *HOXA* gene expression, whereas both *HOXA* and *HOXB* genes are expressed in *NPM1*-mutant AML cells. *HOXA* and *HOXB* genes are simultaneously downregulated during normal myeloid maturation [1], which implies that the clusters may be controlled by common factors that may function in different ways in *NPM1*-mutant vs. *MLL*-rearranged AML cells. We also observed differences in *HOXA* cluster expression patterns in MOLM13 vs. OCI-AML3 cells, including higher expression of *HOXA11*, *HOXA13*, and the *HOTTIP* long noncoding RNA in OCI-AML3 cells. *HOTTIP* has an established role in *HOXA* gene regulation [34, 35], and its higher expression may influence the requirement for certain CTCF-mediated chromatin architectures, thereby making these CTCF binding sites dispensable for steady-state expression of posterior *HOXA* genes in *NPM1*-mutant AML cells.

Three-dimensional architecture is an important component of the regulatory control of *HOXA* genes during development. Our analysis of Hi-C data from OCI-AML3 cells and *NPM1*-mutant primary AML samples identified long-range loops between the active posterior *HOXA* genes *HOXA9*-*HOXA13* and sequences with enhancer-associated epigenetic marks in the *SNX10* and *SKAP2* genes. Additional loops were identified at other loci, but these did not possess active chromatin modifications in primary AML samples. AML cells without *HOXA* expression did not display the same interactions, and lacked active histone marks at the interacting loci, which provides evidence that these contacts may be functionally relevant for *HOXA* gene expression in AML. Interestingly, loss of CTCF within the *HOXA* cluster revealed the plasticity of these interactions, implying that multiple loci may serve as exchangeable *HOXA* enhancers, which is reminiscent of other well-studied, complex regulatory systems, and has been implicated in developmental *HOXA* gene regulation [36, 37].

Our data showed that transcriptional activity and active histone modifications remained highly localized to the posterior *HOXA* cluster in OCI-AML3 cells, even after elimination of key CTCF boundaries. In fact, there was a tendency for deletion mutants to show increased *HOXA* gene expression levels compared with wild-type cells, which is consistent with recent evidence suggesting that reduction of CTCF levels could enhance *HOXA* gene expression [38]. These observations further suggest the posterior *HOXA* cluster possesses intrinsic properties that maintain its active state. It is unclear whether this activity is caused by the compensatory interactions we observed, or if these chromatin loops are consequences of persistent gene expression that is mediated by autonomous, sequence-specific factors that recruit transcriptional machinery. The *HOXA* cluster contains many highly conserved noncoding elements beyond CTCF binding sites. Additional studies will be required to better understand whether these elements provide key signals that promote and maintain *HOXA* gene expression in *NPM1*-mutant AML cells.

## Supplementary information

Supplemental Figures and Figure Legends

Supplemental Table S1

Supplemental Table S2

Supplemental Table S3

Supplemental Table S4

Supplemental Table S5

## Data Availability

All processed sequence data from this study (e.g., bigwig files for ChIP-seq, TPM values for RNA-seq, and Hi-C contact matrices) are available for public download at the following site: https://wustl.box.com/v/ghasemiCTCFHOXA. Raw sequence files for cell lines are available upon request.
